# Breast Cancer-Initiating Cells: Insights into Novel Treatment Strategies

**DOI:** 10.3390/cancers3011405

**Published:** 2011-03-16

**Authors:** Guido Santilli, Mara Binda, Nadia Zaffaroni, Maria Grazia Daidone

**Affiliations:** Department of Experimental Oncology and Molecular Medicine, Fondazione IRCCS-Istituto Nazionale dei Tumori, Via Amadeo 42, Milan 20133, Italy; E-Mails: guido.santilli@istitutotumori.mi.it (G.S.); mara.binda@istitutotumori.mi.it (M.B.); nadia.zaffaroni@istitutotumori.mi.it (N.Z.)

**Keywords:** breast cancer-initiating cells, self-renewal pathways, survival pathways, prognosis, drug resistance, drug targets

## Abstract

There is accumulating evidence that breast cancer may arise from mutated mammary stem/progenitor cells which have been termed breast cancer-initiating cells (BCIC). BCIC identified in clinical specimens based on membrane phenotype (CD44^+^/CD24^−/low^ and/or CD133^+^ expression) or enzymatic activity of aldehyde dehydrogenase 1 (ALDH1^+^), have been demonstrated to have stem/progenitor cell properties, and are tumorigenic when injected in immunocompromized mice at very low concentrations. BCIC have also been isolated and *in vitro* propagated as non-adherent spheres of undifferentiated cells, and stem cell patterns have been recognized even in cancer cell lines. Recent findings indicate that aberrant regulation of self renewal is central to cancer stem cell biology. Alterations in genes involved in self-renewal pathways, such as Wnt, Notch, sonic hedgehog, PTEN and BMI, proved to play a role in breast cancer progression. Hence, targeting key elements mediating the self renewal of BCIC represents an attractive option, with a solid rationale, clearly identifiable molecular targets, and adequate knowledge of the involved pathways. Possible concerns are related to the poor knowledge of tolerance and efficacy of inhibiting self-renewal mechanisms, because the latter are key pathways for a variety of biological functions and it is unknown whether their interference would kill BCIC or simply temporarily stop them. Thus, efforts to develop BCIC-targeted therapies should not only be focused on interfering on self-renewal, but could seek to identify additional molecular targets, like those involved in regulating EMT-related pathways, in reversing the MDR phenotype, in inducing differentiation and controlling cell survival pathways.

## 1. Introduction

Although advances over the last decades in diagnosis and treatment have resulted, since the early nineties, in decreased mortality, breast cancer remains the most common malignant disease in Western women. Breast cancer still represents a major public health problem, with more than 370,000 new cases and 130,000 deaths per year in Europe in women 35 to 64 years of age, and an overall survival at 5 and 10 years of around 68% and 50%, respectively [1].

In breast cancer patients, rather than the primary tumor, metastases at distant sites are the main cause of death. Although hormone therapy prevents progression of hormone receptor-expressing tumors for several years, most patients relapse, making chemotherapy the sole therapeutic option. However, chemotherapy in the metastatic setting yields 20 to 80% responses for a few months and thus remains palliative [2]. The only recent major significant advance is the emergence of targeted therapies, in particular directed against HER2/neu by using the monoclonal antibody trastuzumab or the receptor tyrosine kinase inhibitor lapatinib. Trastuzumab treatment leads to regression of HER2-expressing tumors (20% of cases) and significantly improves patient survival. Adjuvant therapy may help to eradicate breast cancer cells that have already spread to distant sites by the time of diagnosis. Since it is difficult to accurately predict the risk of metastasis in individual patients, nowadays more than 80% of them receive adjuvant chemotherapy, although only approximately 40% relapse and ultimately die of metastatic breast cancer [3]. Therefore, many women who would be cured by local treatment alone, which includes surgery and radiotherapy, will be ‘over-treated’ and needlessly suffer from toxic side effects of chemotherapy.

Thus, a more effective treatment of breast cancer is urgently needed. In particular, it is important to identify new prognostic markers to accurately establish low-risk and high-risk subsets of patients and innovative therapies to eradicate the metastatic breast cancer cells at the stage of the primary tumor. In this regard, despite its clinical importance, the knowledge of genetic and biochemical determinants of metastases of breast cancer cells is limited. Therefore, the identification of pathways central in disseminated breast tumor cell survival and, consequently, the challenge of targeting these pathways may permit, by restoring sensitivity to apoptosis, senescence and immunosurveillance, the eradication of dormant metastatic breast cancer cells.

The goal to identify and characterize the metastatic precursor cells is closely connected to the extensively discussed concept of cancer stem cells (CSC). Findings obtained in the last few years indicate that breast cancers contain a small population of cells with stem-cell-like properties, which may arise from mutated breast stem/progenitor cells that retain the ability to form new tumors when a few cells are transplanted in immunodeficient mice [4]. Breast cancer stem/progenitor cells, also designated as breast cancer-initiating cells (BCIC), display competence for cell renewal and differentiation, like human mammary stem/progenitor cells [5]. Moreover, molecular signatures indicating “stemness” or derived from stem cell-like BCIC showed an association with disease progression [6,7]. A putative cell stem-like phenotype proved to be expressed by early disseminated breast cancer cells that were detected in bone marrow [8]. However, notwithstanding the demonstration that distinct cancer cell subpopulations of breast cancer do express stem cell-like markers, very recent data have questioned the validity of the CSC hypothesis in breast cancer and rather supported clonal heterogeneity as the underlying reason for the tumor cell hierarchy [9].

Currently, projects addressed to identify, isolate and characterize BCIC, aim to elicit genes/signatures associated with factors and signaling pathways involved in regulation of the self-renewal program of mammary stem cells. Such investigations propose to establish whether these genes are detectable in clinical tumors and pre-neoplastic lesions, are associated with tumor progression in different clinical situations, and/or may provide novel therapeutic targets to specifically interfere with BCIC properties. The studies generally exploit clinical models, such as surgical/biopsy specimens for translational studies, taking advantage of collections of formalin-fixed, paraffin-embedded preneoplastic and neoplastic breast lesions and/or frozen breast cancer specimens from patients entered in treatment protocols. In addition, preclinical studies are carried out on *in vitro* cultures of stem-like, tumor-initiating cells from established human breast cancer cell lines (representative of the main breast cancer phenotypes) and clinical tumors isolated on the basis of the expression of putative “stemness markers” and/or growing as nonadherent spheres and on engraftment of clinical tumors directly into immunocompromized mice, to propagate *in vivo* highly tumorigenic cells. Such approaches are instrumental for the functional validation of new targets identified on clinical tumors through analysis of the consequences of their down-regulation by specific inhibitors on breast cancer stem/progenitor cell properties and for assessing the effect of epigenetic modulation-based approaches on self-renewal capacities. Hence, such studies should increase knowledge of the molecular mechanisms involved in breast tumor initiation and progression and determine the relevance of particular signal transduction pathways to self renewal, in order to relate the presence of breast stem/progenitor cell phenotypes to existing subclassifications of breast cancer and to patient outcome and to identify novel therapeutic targets.

We reviewed studies published in the last few years addressed to investigate the biological properties of BCIC, the role of stemness-related markers on breast cancer progression, and the relevance of BCIC features/experimental systems for cancer therapy, in terms of association with clinical response to systemic treatments and identification of new potential therapeutic targets.

## 2. Isolation of Breast Cancer Cells with Stemness Features

Distinct phenotypic and functional assays are currently used to isolate and characterize CSC and to define their frequency in the different tumor types. BCIC are termed cancer cells with high tumorigenicity and self-renewal capability, as assessed by injecting them at very low concentrations (100–1,000 cells) into immunocompromized mice, and have been prospectively identified in clinical specimens and in breast cancer cell lines based on different approaches. Such approaches include analyses of the expression of surface markers, enzymatic activity, and the capability to exclude vital dyes (such as Hoechst 33342 or rhodamine-123, which is exhibited by the so-called side population), and growth as non-adherent spheres (mammospheres) [5]. However, the specificity and reliability of the markers and functional assays used to identify and isolate BCIC are currently under evaluation because of non-univocal data among laboratories and non-overlapping results using the different approaches on the same cell populations, and an accurate and widely accepted definition of markers and assays is therefore mandatory in this field of research. In fact, the relative frequency of BCIC may vary as a function of the specific experimental system and the approach used to isolate and propagate these cancer cells, and their absolute frequency is highly dependent on experimental conditions. Moreover, large-scale analysis of BCIC marker expression should be undertaken to establish whether BCIC share a common phenotype or display variability in marker expression.

### 2.1. Expression of Surface Markers

The seminal study by Al-Hajj *et al.* [4] showed that CD44^+^/CD24^−/low^/lin^−^ (lack of expression of CD2, CD3, CD10, CD16, CD18, CD31, CD64 and CD140b) breast cancer cells obtained mainly from pleural effusions could form tumors when as few as 200 were injected into the mammary fat pad of nonobese diabetic (NOD)/severe combined immunodeficient (SCID) mice. In patients with breast cancer, the presence of CD44^+^/CD24^−/low^ cells did not provide clinically relevant prognostic information notwithstanding the finding that the fraction of CD44^+^/CD24^−/low^ cells was higher in tumors developing distant metastasis than in those that did not [10] and was detected in 50/50 bone marrow specimens with cytokeratin-positive cells, that is, in the so-called disseminated tumor cells [8]. Moreover, compared to normal breast epithelium, they showed a signature accounting for 186 differentially expressed genes (invasiveness gene signature, IGS) mainly involved in the IκB/NFκB (nuclear factor of kappa light polypeptide gene enhancer in B-cells 1) and RAS/MAPK (mitogen-activated protein kinase) pathways and in the epigenetic control of gene expression. This signature was predictive of clinical outcome not only in breast cancer, but also in lung and prostate cancers and in medulloblastoma [7]. Among cell surface markers, also the expression of CD133, a putative stem cell marker for tumor types of different origin, has been reported to identify breast cancer cell subpopulations endowed with tumor-initiating capability [11].

### 2.2. Enzymatic Activity

A recently proposed and currently very popular approach used to identify BCIC is based on enzymatic activity of aldehyde dehydrogenase 1 (ALDH1), a detoxifying enzyme responsible for metabolization of aldehydes and oxidization of retinol to retinoic acid and related to the stemness-related markers Oct-4 and BMI-1 and proven to label stem/progenitor cells in neural and hematopoietic systems and in the mammary gland [12]. Use of the ALDEFLUOR approach (by flow cytometry) and/or the detection of ALDH1-positive cells at the cytoplasmic level by immunohistochemistry identified highly tumorigenic cells not only within breast cancer specimens but also in brain tumors, leukemia and multiple myeloma. In breast cancer, the fraction of ALDH1-positive cells correlates with the molecular subtypes, being highly expressed in the basal-like and HER2 subtypes, but not with other patho-biologic features [13] and is predictive of clinical outcome in breast cancer series in different clinical settings [12,14], even if such a finding is not univocal [13].

### 2.3. Side Population

The capability to exclude vital dyes, such as Hoechst 33342 or rhodamine-123, is characteristic of cells overexpressing transmembrane transporters, like the ATP-binding cassette (ABC) molecule ABCG2/BCRP (ATP-binding cassette, sub-family G/breast cancer resistance protein-1), and might identify cells accounting for the so-called side population. In breast epithelium, side population cells are limited in number (0.5–3%) and are endowed with stem cell properties [15]. Moreover, side population cells are also reported to exist in breast cancer and mainly in human breast cancer cell lines [16]. Notwithstanding the proper identification of the side population still represents a controversial issue, the “side population” cancer cells proved to be resistant to toxins and drugs used in anticancer treatment and to exhibit a higher tumor-initiating capability than non-side population cells, and have been suggested to be enriched in CSC [17].

### 2.4. Mammosphere Formation

BCIC have also been isolated and *in vitro* propagated for some passages as non-adherent spheres of undifferentiated cells (mammospheres) in medium without serum and supplemented with growth factors. The culture of breast cancer cells as mammospheres is a relatively new technique based on methods used for growing neurospheres and recently applied by Dontu *et al.* [5,18] also to mammary epithelial cells to enrich mammary stem cells. We adopted such an approach to propagate *in vitro* as non-adherent mammospheres breast tumorigenic cells with stem/progenitor cell-like properties isolated from clinical tumors as well as from established cancer cell lines (MCF-7 and more recently, 734B and estrogen receptor (ER)-positive and ER-negative human breast xenografts transplanted in nude mice). Mammosphere-derived breast cancer cells display self-renewal properties, overexpress stemness (Oct4 and BMI1) and cytoprotective markers (the IAP survivin protein) and telomerase activity, and are highly tumorigenic when injected at low concentrations in immunocompromized mice [19].

The possibility to propagate *in vitro* BCIC using a functional approach, such as the sphere-forming assay, might represent a potentially valid and convenient tool to enrich cell cultures for stem cell-like cancer cells, in order to molecularly characterize and challenge them against conventional, investigational and novel therapies targeting pathways biologically relevant for CSC. However, the yield of BCIC from primary tumors is highly variable and often contaminated by more differentiated cells, mainly when the number of BCIC is modest, and culture conditions may be suboptimal and not adequate to support their propagation, since after a few *in vitro* passages, induction of differentiation and/or senescence results in loss of stemness.

### 2.5. Stem Cell Patterns in Human Cancer Cell Lines

Side population, expression of surface markers, ALDH1 activity and capability to form non-adherent spheres have also been recognized in cancer cell lines that have been established from different solid tumor types [17]. Stem cell-like cancer cells isolated by distinct approaches from established breast cancer cell lines have been considered as surrogate models of cultures of tumor-initiating cells derived from clinical tumors (which are difficult to obtain and *in vitro* successfully propagate for high-throughput screening of lead compounds or drug development, which in turn requires a large number of stem-like cells) or of direct xenografts of surgical tumor specimens in immunocompromized animals (which are successful in less than 20% of breast cancers). However, much effort should be made to assess the reliability of the system after several *in vitro* passages, as well as its independence of experimental conditions and its genetic stability. In fact, in human breast cancer cell lines, the propagation of non-adherent spheres, which are endowed with high tumorigenicity, rather than adherent cells, may result in a more rearranged karyotype, and display additional and more complex chromosomal rearrangements [20], which puts into question their use for high-throughput screening.

Our experience with breast cancer cells obtained from established cell lines, clinical tumors and pleural effusions, and xenografts of human tumors, indicates that a common, distinctive feature of stem cell-like breast tumor-initiating cells is currently not available. Indeed, the CD44^+^/CD24^−/low^ status commonly used to identify tumor-initiating breast cancer cells is present in a substantial fraction of cells derived from pleural effusions or from ER-negative breast cancer cell lines and xenografts, whereas it is detectable in a lower fraction of cells from ER-positive tumors, even growing as non-adherent mammospheres. Conversely, other putative stemness markers, such as the presence of a side population expressing the Hoechst 33342 dye or ALDH1, were singly detectable in breast cancer cell lines or xenografts that rarely produce mammospheres [21].

## 3. Signaling Pathways Involved in the Regulation of Self-Renewal of BCICs

Several signaling pathways known to be crucial for stem cells and development are proven to be involved in cancer, including those of epithelial origin. They include Wnt (wingless-type MMTV integration site family), Notch, Nanog, Oct-4, hedgehog and BMI-1 (B lymphoma Mo-MLV insertion region 1 homolog) signaling pathways. In addition, other signaling pathways relevant for BCIC are those of integrins [22], insulin-like growth factor-1 [23], ER and progesterone receptors (PgR) [24], epidermal growth factor (EGF)-like/EGF receptor (EGFR) and HER2/Neu [24], BRCA-1 (breast cancer-1) [26], leukemia inhibitory factor [27], SDF-1/CXCR4 (stromal cell-derived factor 1/chemokine receptor 4) [10], interleukin-6 [28]. Such signal interplay contributes to generate the unique features of breast tumor-initiating cells, which include self-renewal, proliferation, differentiation and survival. In addition to self-renewal properties, breast tumor-initiating cells display epithelial-mesenchymal transition (EMT) [29], a mechanism physiologically involved in development and tissue remodeling but pathologically dealing with progression of different diseases, including inflammation and cancer. The interaction between self renewal and EMT properties may account for the mechanism supporting the metastatic process and growth in distant sites of cancer lesions morphologically similar to the primary tumors. A distinct family of miRNA, the let-7, has been demonstrated to link tumor-initiating cells with EMT through the regulation of several stemness-related pathways and the silencing of multiple genes [30]. Also cell survival factors, such as telomerase and antiapoptotic proteins (survivin and Bcl-2), and pro-angiogenic factors (vascular endothelial growth factor) proved to be activated/overexpressed in BCIC [19] and may represent additional targets for treatment strategies. Preliminary findings in clinical breast cancer ascribe a prognostic role to some components of self-renewal pathways. We review here some clinically relevant information obtained from investigations of these pathways in clinical breast cancers and experimental preclinical models.

### 3.1. Wnt Signaling

Initially implicated in mammary cancer development and maintenance of the stem cell/progenitor pool in the mouse mammary gland, a Wnt cross-talk with steroid receptor pathways has been recently shown, and Wnt signaling down-regulation by Wnt antagonists or small interfering RNAs (siRNA) proved to increase the expression of differentiated markers in human breast cell lines [31]. This suggests that the molecular targeting of Wnt signaling could represent a potential therapeutic approach. In clinical breast cancer, contrasting results have been reported for the association between Wnt5a expression and prognosis [32–34], whereas intracellular β-catenin expression localization [35] is differently associated with clinical outcome (favorable for cytoplasmic, unfavorable for nuclear), and promoter methylation of the secreted frizzled-related protein 1 appears as an independent factor for adverse patient survival [36,37].

### 3.2. Oct-4 Signaling

The Oct-4 transcription factor plays a pivotal role as a key regulator of pluripotency in the earliest stages of mammalian development. Because of its ability to maintain the stem cell state and thus to be refractory to xenobiotic agents, it was hypothesized that Oct-4 aberrant expression may contribute to the activation and maintenance of neoplastic process in normal and tumor cells [19,38]. A possible role for Oct-4 in tumorigenesis is supported by several additional lines of evidence and it is therefore desirable to develop experimental molecules capable of selectively targeting this pathway.

### 3.3. Nanog Signaling

Nanog is a newly identified homeodomain-bearing transcriptional factor and it represents a key molecule involved in the signaling pathway for maintaining the capacity for self-renewal and pluripotency. Several studies have suggested that the molecular stem-maintenance circuitry controlled by Nanog may be active in breast tumors. Thus it is one of the molecular markers suitable for recognizing and targeting the undifferentiated state of cells in malignant human tissue [38].

### 3.4. Hedgehog Signaling

Hedgehog cascade has been implicated in the development of several cancers including breast cancers. Sonic hedgehog contributes to a molecular signature segregating inflammatory breast cancers at different prognosis [39], whereas exposure to cyclopamine, an alkaloid blocking hedgehog pathway, suppresses Gli1 expression and breast cancer cell growth [40].

BMI-1 is a component of the Polycomb complex induced through the hedgehog signaling pathway, which is responsible for self renewal of normal and leukemic stem cells, represses genes inducing cell senescence and death, and immortalizes human mammary epithelial cells. BMI-1 is overexpressed in breast cancer cells growing as non-adherent mammospheres [41], and a BMI-1-based 11-gene signature is a powerful, therapy-independent predictor of recurrence, distant metastasis and death in 11 epithelial and non-epithelial cancers [6]. Such findings suggest that the targeting of BMI-I could also constitute a potential therapeutic strategy.

### 3.5. Notch Signaling

Notch cascade is known to play a role in several cancers [42,43], and Notch 1 and 4 are involved in tumor induction in mouse and in increased proliferation in mammospheres [44,45]. Notch 1 and 2 are directly and inversely associated to an unfavorable outcome in breast cancer patients, respectively [44–47].

### 3.6. HER-2 Signaling

HER-2, a member of the EGFR family whose amplification is present in about 25% of human breast cancers, correlates with a distinct molecular profile and unfavorable outcome and has been shown to regulate the mammary stem/progenitor cell population driving tumorigenesis and invasion [25]. In fact, in normal mammary epithelial cells, HER2 overexpression increases the proportion of stem/progenitor cells (*in vitro* mammosphere assay), the expression of the stem cell marker ALDH1, and the generation of hyperplastic lesions in the humanized mammary fat pad of NOD/SCID mice. Similarly, in breast carcinoma cell lines, HER2 overexpression increases the ALDH-expressing BCIC population, which displays enhanced expression of stem cell regulatory genes and exhibits increased invasion *in vitro* and increased tumorigenicity in the humanized mammary fat pad of NOD/SCID mice [48].

### 3.7. EMT

Epithelial to mesenchymal transition is an essential process that enables reprogramming of polarized epithelial cells towards a mesenchymal motile phenotype during physiological remodeling in normal adult tissues. In fact, the typically dormant EMT program is reactivated during wound repair and tissue regeneration. Recent studies have demonstrated that EMT is associated with the expression of many stem cell markers and phenotypes in human mammary epithelial cells in parallel with loss of E-cadherin expression, a key component of adherent junctions [49]. These studies suggest that induction of EMT by extracellular stimuli and microenvironment factors could confer both self-renewal and metastatic properties to breast cancer cells resulting in *de novo* generation of cancer stem cells population from differentiated tumor cells, thus making this cellular mechanism an alternative driving force in tumor progression. Overall, the findings that link EMT and cancer stem cell hypothesis indicate that targeting both pathways simultaneously may hold considerable therapeutic promise [29,49].

## 4. Stemness-Related Signature and Biomarkers Associated with Tumor Progression

Invasiveness gene signature (IGS), derived from the comparison between CD44^+^/CD24^low/−^ tumorigenic breast cancer cells and normal breast epithelium, has been recently reported to be associated with metastasis-free survival irrespective of treatment and in patients receiving only local-regional treatment [7]. It appears to be overexpressed in basal-cell breast cancer, likely ER-negative, although its prognostic role is mainly evident within ER-positive cancers. Since no information was provided about its prognostic role within an antiestrogen treatment setting, we challenged IGS in a series of 110 postmenopausal patients treated with radical or conservative surgery plus radiotherapy and submitted to adjuvant monotherapy with tamoxifen (40 mg daily) for at least three years (according to the clinical practice in use at our Institute in the period 1991–1996), who relapsed during adjuvant treatment or resulted disease-free over 70 months of follow-up [50]. We profiled these tumors with cDNA microarrays, which included 76 of the 186 genes defining the IGS. When patients were stratified according to the correlation of their gene expression pattern with IGS, we observed that about 70% of disease-free cases had tumors with a low IGS (*P* = 0.026). This observation was also confirmed by univariate survival analysis, which indicated a higher hazard ratio (HR) for relapse (HR, 1.89; 95% CI, 1.05–3.38, *P* = 0.033) in patients whose tumor gene expression pattern showed a direct association to IGS than that of patients whose transcriptional profile showed a strong inverse association to IGS. Additionally, IGS was able to further discriminate, among the 83 patients with PgR-positive tumors, those with the worst prognosis.

In conclusion, although our gene expression data base included only about 40% of the IGS genes, our results indicate IGS as a predictor of metastasis also in ER-positive breast cancer patients treated with adjuvant tamoxifen and possibly, since it appears able to further identify patients with the worst outcome even for PgR-positive tumors, it may be associated to anti-estrogen resistance.

In this series of ER-positive early stage breast cancers, gene expression profiling allowed us to obtain information on the expression of a panel of genes, putatively associated to stem cell phenotypes. These genes include AKT1 (v-akt murine thymoma viral oncogene homolog 1), ALDH1, AXIN1 (axis inhibitor 1), BMI-1, CCND3 (cyclin D3), GAS1 (growth arrest-specific 1), GLI1 (glioma-associated oncogene family zinc finger 1), GLI3, MSI1 (musashi homolog 1), MSI2, MYC, NUMB, PDK1 (pyruvate dehydrogenase kinase, isozyme 1), PTCH (patched homolog 1) receptor and WNT4. In addition, we considered GATA3 as an indicator of a typical luminal phenotype that in the mammary gland has been described to drive a stem cell-enriched population along the alveolar luminal lineage and to play a role in breast tumorigenesis. Overall, these genes proved to be co-expressed within the specific pathway (WNT, BMI, Notch, sonic hedgehog), but their expression was generally independent among pathways and only weakly associated with clinico-pathologic (patient age, tumor size and lymph node involvement) and biological findings (ER, PgR and HER2). The only exception to these findings was represented by GATA3, which, as expected, was positively correlated only with ER mRNA and protein concentration (rs = 0.34, *P* = 0.0002 and rs = 0.35, *P* = 0.0002, respectively). In this case series, patient age, lymph node involvement and PgR status were significant predictors of relapse-free survival at seven years of follow-up, whereas large tumor size and high histological grade were only suggestive of an unfavorable prognosis. We observed that an altered expression of genes involved in stem cell renewal pathways (sonic hedgehog, PTEN and BMI) proved to be associated with new disease manifestations (Table 1).

Also, their prognostic relevance increased when associated to alterations in developmental pathways (homeobox family and GATA3). In this case series, ALDH1 was also positively associated with development of metastasis. Such findings related to stem cell- and developmental pathway-related genes, obtained in a clinically homogeneous although selected (since ER-positive) breast cancer subset, indicate a potential for self renewal within tumors and for increased tumor aggressiveness within patients and provide evidence that stemness-related properties may partly explain endocrine resistance in breast cancer patients.

**Figure 1.**
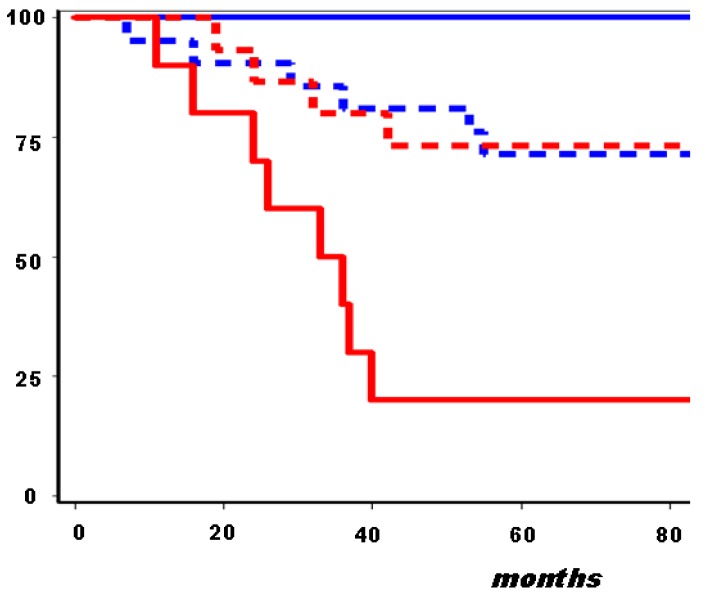
Probability of relapse-free survival (%) according to Gata3, PTCH1 and ALDH1 expression in 110 node-positive, ER-positive resectable breast cancers from postmenopausal patients treated with adjuvant tamoxifen. Blue solid line, patients with tumors presenting with all favorable markers (18 cases, 0 unfavorable events); broken lines, patients with tumors presenting with 1–2 unfavorable markers (72 cases, 22 unfavorable events); red solid line, patients with tumors presenting with all unfavorable markers (20 cases, 16 unfavorable events).

Similar findings were also validated from the analysis of public data bases (node-negative breast cancers from the Netherlands Cancer Institute [51]), which confirmed the prognostic value of an altered expression in self-renewal pathways, including hedgehog (Gli3 e PTCH1), Wnt (FZD6), and Notch (Jag2). Moreover, in our case series of ER-positive breast carcinomas, the combined consideration of GATA3, ALDH1 and PTCH1 allowed the identification of two subsets of patients at minimal risk (18 cases disease free 7 years after the diagnosis) or at a very high risk of relapse (16 out of 20 cases with new disease manifestations, mainly in distant sites) (Figure 1).

An integration approach coupling transcriptome analysis of clinical tumors with preclinical interference of progression-related alterations in pathways critical for self renewal may help to translate the outcome of BCIC biology into clinically useful information with the identification of novel diagnostic markers and therapeutic targets.

## 5. Implication of BCICs in the Response to Systemic Treatments

The concept that cancer may be a stem cell disease, arising from tissue stem/progenitor cells or driven by cancer cells with stem cell properties, has important implications for understanding the basic biology of tumorigenesis and is crucially relevant to cancer management, for the development of new strategies for prevention, staging/prognosis and therapy [52–54]. In fact, current therapeutic strategies aimed at reducing tumor size, while producing dramatic volume reduction, are unlikely to result in long-term remissions if they do not target the limited fraction of cancer stem/progenitor cells including BCIC, which share many properties of normal stem cells that provide resistance to drugs and toxins. Integrated treatment approaches and appropriate means to evaluate treatment efficacy early should also be investigated, since tumor shrinkage, commonly considered as an indicator of clinical response, may leave unaffected the BCIC, which can again proliferate, leading to relapse. Conversely, treatments selectively targeting BCIC but failing and/or lessening the elimination of differentiated tumor cells might be prematurely abandoned if clinical activity is evaluated by traditional response criteria reflecting changes in the bulk of the tumor.

### 5.1. Correlative Studies in Clinical Tumors

The resistance of tumor-initiating cells to anticancer drugs has been hypothesized based on their unique properties to reduce drug uptake and effect because of repair and anti-immune mechanisms, which are in keeping with inherent resistance to a variety of conventional agents. Such findings have been shown in different *in vitro* experimental systems and indirectly by the retrospective observation that residual post-treatment tumors are enriched for cells with tumor-initiating capability [30]. Importantly, the intrinsic resistance of BCIC to chemotherapy has been recently directly demonstrated also on a series of 31 primary breast cancers submitted to neoadjuvant chemotherapy with docetaxel or doxorubicin/cyclophosphamide [55]. Chemotherapy treatment increases the fraction of CD44^+^/CD24^low/−^ and the mammosphere formation efficiency in residual tumor cells after treatment, thus providing strong evidence from a clinical setting for a subpopulation of chemotherapy-resistant BCIC. Conversely, in a parallel series of HER2^+^ tumors from 21 patients treated with lapatinib, the residual tumor cell population following neoadjuvant treatment did not show an increase in tumor cells with putative stem cell features, thus providing support for the hypothesis of breast cancer stem cells, since HER2 is a BCIC driver and the evidence that specific inhibitors of signaling pathways involved in self renewal (EGFR/HER2) may provide a therapeutic strategy for eliminating BCIC [25].

Another analysis carried out on a series of 108 primary breast cancer patients treated with neoadjuvant therapy consisting of sequential paclitaxel and epirubicin-based regimens has not indicated an association between increased fraction of CD44^+^/CD24^low/−^ cells and chemotherapy resistance but showed ALDH1 overexpression in non-responding tumors as well as in the residual tumor cell population [54]. Other studies challenged the role of a stem-cell phenotype (as defined by the fraction of cells CD44^+^/CD24^−^ or expressing ALDH1) in tumor resistance to chemotherapy. In fact, in a series of 66 breast cancers subjected to primary systemic treatment with regimens including epirubicin/cyclophosphamide or doxorubicin/pemetrexed, the post-treatment residual tumor cell population was not enriched by CD44^+^/CD24^−^ cells [57]. Similarly, a recent investigation on a substantial series of neoadjuvantly treated cases (with paclitaxel and epirubicin-based regimens plus herceptin if HER-positive tumors) and triple-negative breast cancers did not confirm the association between ALDH1 expression in tumor cells and clinical outcome, whereas expression of ALDH1 in stromal cells was increased following neoadjuvant treatment and was significantly associated with disease-free and overall survival in the triple-negative cases. These observations suggest a possible role of the tumor microenvironment in affecting the prognostic relevance of putative stemness markers [13].

We also analyzed the expression of ALDH1 and CD44^+^/CD24^low/−^ in a series of 20 matched primary tumors/metachronous metastasis (mainly from soft tissues). The analysis showed an increased frequency of CD44^+^/CD24^low/−^ cases from the primary to the metastatic lesion [58].

The non-univocal results in these preliminary translational studies emphasize the importance of an experimental design requiring a large number of clinical specimens (possibly derived from patients entered in clinical trials, to improve the level of evidence of results) from tumors homogeneous for stage and biomolecular subtype and of standardized technical and analytical approaches.

### 5.2. Therapies Targeting BCICs

Recent findings in different tumor types indicate that the combined targeting of molecules of self renewal and developmental pathways, associated with conventional agents, could represent a promising treatment strategy in patients with advanced disease [59,60]. Several lines of evidence support these findings in experimental preclinical systems in which targeting such key molecules may interfere with stemness hallmarks even in malignant stem-like tumorigenic cells. *In vivo* assays still represent the gold standard for identifying and studying novel therapeutic approaches against CSC, but they are time consuming (several months are needed for serial transplantation experiments) and thus make a high-throughput screen to validate compounds difficult. In addition, for some tumor types like breast cancer, the taking of a direct engraftment of tumor specimens into immunocompromized mice is too low to allow for a large-scale screening of novel therapeutic agents. Conversely, the propagation of *in vitro* cultures of BCIC represents a challenge for dissecting the effect of investigational agents directly on stem cell biology and an opportunity for the possibility to also investigate differentiation as an end point. However, it does not guarantee unbiased results, for being “niche-independent” since interactions with the tumor microenvironment are generally not considered in the assay conditions, as well as for the necessity to control for genetic instability, which might be induced by serial *in vitro* cell propagation cycles. Indeed, additional and more complex chromosomal rearrangements have been observed in cancer cells from non-adherent spheres compared to the parental populations [20]. Moreover, *in vitro* assays—which are quantitative and rapid—should ideally also be specific, measuring only the cells of interest, and sufficiently sensitive to measure candidate CSC when present at a low frequency. The possibility to couple *in vitro* assays and *in vivo* injection of cells in immunocompromized mice might overcome limitations related to each single test and provide mechanistic insight into the molecular cross-talks, while still representing a valuable pre-clinical system for screening and validation of novel therapeutic approaches.

In the last few years, BCIC isolated on the basis of the capability to grow as non-adherent spheres or the presence of stemness-related markers (CD44 /CD24^low/−^ and ALDH1, side population) from cell lines and clinical specimens have been challenged against different molecules to investigate the potential of directly targeting BCIC for new therapeutic strategies (Table 2). We reviewed the inhibitors of self-renewal pathways relevant to breast cancer development and differentiating agents as potential strategies to target BCIC and their progenies.

### 5.3. Potential Therapeutic Targets in BCICs

It has been shown that therapeutic approaches targeting the Notch pathway (both selectively, using monoclonal antibodies or RNA interference, and non-selectively, using inhibitors of enzymes involved in glycosylation) were able to suppress the self-renewal ability of BCIC. Specifically, the mammosphere-forming efficiency of non-adherent cultures derived from *in situ* ductal carcinomas was found to be reduced following treatment with a Notch4-neutralizing antibody or the γ-secretase inhibitor DAPT [47]. Similar results were obtained in mammosphere cultures derived from HER-2 overexpressing carcinoma cell lines following siRNA-mediated Notch1 silencing or γ-secretase inhibitor I (GSI) treatment [48]. It has also been reported that clinically used inhibitors of receptor tyrosine kinases such as gefitinib, a small molecule inhibitor of EGFR, and trastuzumab, the humanized monoclonal antibody directed against HER-2, were also able to reduce tumor-sphere-forming capability [25,47,48]. In addition, the loss of serial transplantability was also reported following treatment of HER-2 overexpressing xenotransplants with trastuzumab [48].

The possibility of specifically targeting self-renewal ability of cancer stem cells led to the use of Suberoylanilide Hydroxamic Acid (SAHA), a pan-histone deacetylase (HDAC) inhibitor [61]. Much evidence suggests that HDAC enzymes control reversible chromatin remodeling, which is a key epigenetic mechanism that regulates transcription of genes associated with multiple activities of normal and malignant cells, including self-renewal, survival and resistance to apoptosis, cell cycle progression, aggregation, motility, invasion and metastasis. SAHA inhibited self-renewal of tumor spheroids from established breast cancer cell lines and pleural effusion aspirates from patients with advanced breast cancers, as assessed by decreased clonogenic growth, and markedly affected mammosphere formation and their 3-dimensional structure, which was associated with translocation of E-cadherin protein from the plasma membrane to the cytoplasm [61].

Interestingly, the lentivirus-mediated reconstitution of let-7, a family of miRNAs regulating several stemness- and EMT-related pathways, was able to reduce proliferation, tumor-sphere formation and the fraction of undifferentiated BCIC *in vitro*, as well as to affect tumor and metastasis formation in NOD/SCID mice [30]. Increased let-7 was paralleled by the reduction of two known let-7 targets, such as RAS (important for self renewal) and HMGA2 (high mobility group AT-hook 2, important for multipotency). This suggests the possibility of a therapeutic use of let-7 mimics with minimal toxicity to normal tissues that already express this miRNA family [30].

By coupling gene expression analysis with functional studies, Ginestier *et al.* [62] showed that ALDH1-expressing breast cancer cells also overexpress the IL-8 receptor CXCR1, whose blocking by a specific antibody and/or by the small molecule inhibitor repertaxin reduced the fraction of cells with stemness features in the ALDH1-positive subpopulation from human breast cancer cell lines. This effect was followed by an induction of apoptosis also in the bulk population via a bystander effect mediated by FASL/FAS. In association with docetaxel, repertaxin was able to delay tumor growth and development of metastasis in NOD/SCID mice, thus suggesting novel strategies based on the interference with cytokine regulatory loops to target BCIC. Another study demonstrated that treatment of the side population of breast cancer cell lines with quinoline compound dofequidar fumarate (able to reverse the multidrug resistance phenotype by inhibiting the function of the ABC transporter ABCG2/BCRP) reduced the fraction of cells with stemness properties and sensitized these cells to treatment with irinotecan *in vitro* and following xenotransplantation into nude mice [63].

The possibility to induce differentiation of breast cancer cells characterized by stemness features was demonstrated by treatment with the potassium ionophore salinomycin [64]. Specifically, treatment of mice with the compound was found to inhibit mammary tumor growth and to induce increased epithelial differentiation of tumor cells. The experimental model used to investigate salinomycin activity is of utmost interest in the field of assays challenging the activity of compounds on putative BCIC, which accounted for major concerns either in *in vitro* (with the lack of standardized approaches to isolate and propagate cancer stem cells) and *in vivo* testing. Indeed, the human cancer cells implanted into immunocompromized mice might not realistically recapitulate what happens in patients during cancer initiation and progression. Conversely, the approach proposed by Gupta *et al.* [64] exploits the use of telomerase-immortalized human mammary epithelial cells, in which targeting E-cadherin by short hairpin RNA promotes EMT and the expression of cellular/molecular features characterizing BCIC. These genetically modified cells, and their parental cell line that does not exhibit stemness-related features, represent an experimental model ideally suitable for the high-throughput screening for compounds specifically targeting BCIC. By using this approach, the authors were able to demonstrate that salinomycin reduces the proportion of BCIC by more than 100-fold relative to a conventional anticancer agent such as paclitaxel [64].

Recent findings also provided support to the possibility to develop chemopreventive strategies targeting breast stem cells in high-risk subjects or BCICs. In fact, it has been recently shown that metformin, a standard drug for diabetes, was able to inhibit cell transformation in an inducible model consisting of non-transformed human mammary epithelial cells (MCF-10A) containing ER-src, a fusion of the v-src oncoprotein with the ligand-binding domain of ER, as well as to selectively kill BCIC in three other mammary adenocarcinoma cell lines derived from genetically and phenotypically different tumors [65]. Very recently, it was reported that tranilast, a non-toxic aryl hydrocarbon receptor (AHR) agonist, was able to inhibit mammosphere formation by BCIC from triple-negative breast cancer cells selected *in vitro* for mitoxantrone resistance. The drug was also effective *in vivo* since it prevented lung metastasis in mice injected i.v. with MDA-MB-231 mitoxantrone-selected cells [66]. Again, a novel Gemini vitamin D analogue, BXL0124, was found to inhibit the expression of CD44 in MCF10DCIS.com human breast cancer cells *in vitro* and *in vivo*, as well as to down-regulate the transcriptional activity of the CD44 promoter, suggesting a possible role of the compound for BCIC treatment [67].

Recent studies even on tumor types other than breast cancer, showed the possibility to interfere with CSC properties by novel approaches such as telomerase antagonists [68] and an oncolytic reovirus [69], both active also on tumor bulk.

## 6. Conclusions

The concept that breast cancer may be a stem cell disease, arising from tissue stem/progenitor cells or driven by cells with stem cell properties, has important implications for understanding the basic biology of tumorigenesis. It is crucially relevant to cancer management and the development of new strategies for prevention, staging/prognosis and therapy. Cancer cells with stem-like features exist in breast cancer as well as in most, if not all, tumor types and provide support to a new interpretation of cancer biology in terms of aberrant organogenesis. Experimental evidence is rather solid for the main property (self renewal), and, based on *in vivo* assays, subpopulations of cells endowed with stem-like properties demonstrate high tumorigenic potential. The therapeutic implications of this model system are valuable, since CSC might represent new targets with distinct possible interventional strategies, such as interference with self renewal and CSC phenotype reversal through epigenetic modulation/reprogramming.

However, our understanding of bio-molecular features of CSC in the context of cell biology of breast cancer, and with respect to the corresponding features of normal stem cells, is still fragmentary. In fact, the main CSC hypothesis should also be tested in light of the known genetic heterogeneity and genomic instability of human cancer. Critical molecular alterations characterizing and identifying BCIC, their sensitivity to conventional therapies and the development of resistance to chemical and physical agents, as well as the identification of potential therapeutic targets, represent key questions whose solution may help to translate the outcome of BCIC biology into clinically useful information.

## Figures and Tables

**Table 1.** Risk of metastasis among postmenopausal patients with ER-positive breast cancer treated with radical or conservative surgery plus radiotherapy and adjuvant tamoxifen.

**Variable**	**Hazard Ratio for Metastasis (95% Confidence Interval)**	***P value***
CCNB2 (continuous)	1.8 (1.1–2.9)	0.021
CCND3 (dichotomous, high *vs.* low)	1.8 (1.0–3.2)	0.06
GLI3 (dichotomous, low *vs.* high)	2.1 (1.0–4.4)	0.06
PTCH1 (dichotomous, low *vs.* high)	1.9 (1.0–3.6)	0.04
ALDH1 (dichotomous, high *vs.* low)	3.1 (1.2–7.9)	0.015
HOXB2 (dichotomous, high *vs.* low)	1.6 (1.0–2.6)	0.06
HOXB6 (dichotomous, high *vs.* low)	2.1 (1.0–4.5)	0.04
GATA3 (dichotomous, low *vs.* high)	1.8 (1.0–3.4)	0.05

**Table 2.** Breast cancer-initiating cells and potential therapeutics.

**Experimental model**	**Investigated agents**	**Anticancer effects**	**Reference**
Cells from *in situ* ductal carcinomas (spheres)	EFGR inhibitor (gefitinib), Notch signaling inhibitors (γ-secretase inhibitor [DAPT], Notch4 neutralizing antibody)	– Reduced mammosphere formation	Farnie *et al.* [47]
HER2 overexpressing breast cancer cell lines (ALDH1^+^, spheres)	HER-2 inhibitor (trastuzumab), Notch-1 signaling inhibitors (γ-secretase inhibitor I, Notch 1 siRNA)	– Reduced mammosphere formation – Loss of serial transplantation capability	Korkaya *et al.* [25] Magnifico *et al.* [48]
Breast cancer cell lines and clinical tumors (spheres)	Pan-Histone Deacetylase Inhibitor (suberoylanilide hydroxamic acid)	– Reduced mammosphere formation	Robertson *et al.* [61]
Breast cancer cell lines and clinical tumors (CD44^+/^CD24^−/low^, spheres)	Let-7-lentivirus	– Reduced mammosphere formation – Inhibition of proliferation – Inhibition of tumor and metastasis formation in NOD/SCID mice	Yu *et al.* [30]
Breast cancer cell lines (ALDH1^+^, spheres)	CXCR1-specific blocking antibody, repertaxin	– Reduced fraction of cells with stemness features – Delayed tumor growth and metastasis formation in NOD/SCID mice (when combined with docetaxel)	Ginestier *et al.* [62]
Breast cancer cell lines (side population)	MDR reversing agent (dofequidar fumarate)	– Reduced fraction of cells with stemness features – Increased *in vitro* and *in vivo* sensitivity to irinotecan	Katayama *et al.* [63]
CD44^+/^CD24^−/low^ subpopulation obtained from immortalized mammary epithelial cells undergone EMT	Salinomycin	– Selective killing of BCIC population – Inhibition of tumor growth *in vivo* and metastasis formation – Induction of epithelial differentiation	Gupta *et al.* [64]
Genetically different breast cancer cell lines (CD44^+/^CD24^−/low^, spheres)	Metformin	– Selective killing of BCIC population – Inhibition of tumor growth *in vivo* – Reduced invasion capability	Hirsch *et al.* [65]
Cells from triple negative breast cancer cell lines selected *in vitro* for mitoxantrone resistance (spheres)	AHR agonist (tranilast)	– Reduced mammosphere formation – Prevention of lung metastasis	Prud'homme *et al.* [66]
CD44^+/^CD24^−/low^ human breast cancer cells	Gemini vitamin D analog (BXL0124)	– Reduced expression of CD44 in BCICs *in vitro* and *in vivo* – Reduced transcriptional activity of CD44 protein	So *et al.* [67]
